# Solitary Rectal Ulcer Syndrome: A Clinicopathological Study of 13 Cases

**DOI:** 10.4103/1319-3767.54749

**Published:** 2009-07

**Authors:** Nabeel Al-Brahim, Naser Al-Awadhi, Saleh Al-Enezi, Saqer Alsurayei, Mahmoud Ahmad

**Affiliations:** Department of Pathology, Gastroenterology Unit, Farwania Hospital, Kuwait; 1 Department of Medicine, Gastroenterology Unit, Farwania Hospital, Kuwait

**Keywords:** Constipation, rectal, solitary, ulcer

## Abstract

**Background/Aims:**

Solitary rectal ulcer syndrome (SRUS) is a rare disorder that has a wide spectrum of clinical presentation and variable endoscopic findings. To further characterize the clinical and pathological features, a retrospective, hospital-based clinicopathological study was conducted.

**Materials and Methods:**

All cases of SRUS diagnosed at Farwania Hospital, Kuwait, between 2002 and 2007 were retrieved from the computerized filing system. The histological slides were reviewed by two authors to confirm the diagnosis. Immunohistochemical stain for smooth muscle actin (SMA) was performed. The clinical files were reviewed for clinical features and endoscopic findings.

**Results:**

Thirteen cases were identified: 8 males and 5 females. The age range was 15–85. Rectal bleeding, constipation, and abdominal pain were the most common presenting symptoms and were seen, either alone or in various combinations, in 12 of the 13 cases. Rectal ulceration was the most common endoscopic finding, being seen in 9 of the 13 cases; 3 of these cases had multiple ulcerations. Two patients had rectal polyps, with one of them having multiple polyps. The histological examination revealed surface serration, fibromuscular obliteration of the lamina propria, and crypts' distortion in all the cases. Seven of the cases had diamond crypts. Ectatic mucosal vessels were a common finding. Positivity for SMA in the lamina propria was seen in all examined cases.

**Conclusion:**

SRUS is a rare disorder and only 13 cases were diagnosed in Farwania hospital over a 6-year period. The clinical presentation of our patients was variable. The presence of polyps and multiple ulcerations on endoscopy is further evidence that SRUS is a misnomer. Surface serration, fibromuscular obliteration, and crypts' distortion are the most characteristic features. The presence of diamond crypts is an additional diagnostic feature.

Solitary rectal ulcer syndrome (SRUS) is a rare benign disease of unknown etiology. Despite the name, which suggests that it is a specific entity with typical location and presentation, this is not so. Tjandra and colleagues reported the largest series of SRUS in the literature from Cleveland Clinic.[1] Rectal bleeding, constipation, and straining at stool was the most common presentation, the combination of these symptoms suggesting a local disease in the rectum. However, 20% of the patients presented with diarrhea and 26% were asymptomatic. These findings show that there is a wide spectrum of clinical presentations. This study[1] also showed that the endoscopic findings were variable. Although the name suggests the presence of a solitary ulcer, 50% of the patients had polyps rather than an ulcer and 10% had multiple lesions. The term SRUS is therefore misleading.

The histological examination of the rectal lesion is the key to the diagnosis of SRUS. The characteristic features include surface serration, fibromuscular obliteration, and crypts' distortion.[2–4] In addition, different vascular changes, such as ectasia, congestion, and hyalinization, can be seen.[5] It is important to note that these features are not pathognomonic of SRUS.

This wide spectrum of clinical features, endoscopic findings, and histological features make SRUS a great mimicker of other serious conditions, including adenocarcinoma, inflammatory bowel disease, dysplasia, and adenomatous polyp.[6] Therefore, we conducted this study in order to: 1) further characterize the clinical and pathological features of this syndrome in our population and compare them with the literature reports and to see if geographic location changes the features of the disease; 2) increase the awareness of clinicians and surgical pathologists regarding this entity as it is a great mimicker of other conditions. To the best of our knowledge this syndrome has not been well studied in Kuwait and the Gulf region and ours is the first study of this entity reported from this area.

## MATERIALS AND METHODS

The computer filing system of the Department of Pathology, Farwania Hospital, was searched for the diagnosis of SRUS and rectal ulcer made between 2002 and 2007. The hematoxylin and eosin (H and E) slides were reviewed by two authors (NAB and MA) to confirm the diagnosis. Fourteen cases were identified; one case was excluded because the clinical records were not available for review. The histological features of the cases were further evaluated in detail. Immunohistochemical stain for smooth muscle actin (SMA; 1:50, Dako) was performed on nine cases where there was enough tissue in the block for staining at the indicated dilution. The clinical records were reviewed for details of the clinical presentation, colonoscopic findings, associated local and systemic diseases, and other investigations carried out (e.g., endoanal ultrasonography, anorectal manometry, and pudendal nerve study). The approval of the Ethics Committee of the Ministry of Health was obtained prior to conducting the study

## RESULTS

### Clinical findings

**Figure 1:**
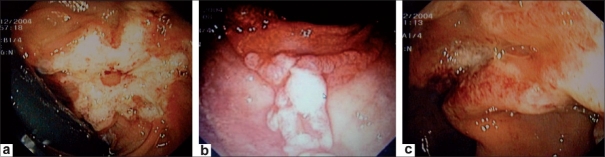
Different colonoscopic appearances of different cases of SRUS: (a) solitary ulcer with irregular borders; (b) multiple polyps; (c) erythematous changes in colonic mucosa

The clinical features and the endoscopic findings are summarized in Table 1. The patients' ages ranged from 15 to 85 years. There were eight male and five females. Rectal bleeding, constipation, and abdominal pain were the most common presentations and occurred, either alone or in various combinations, in 12 (92%) patients. One patient presented with diarrhea. Rectal digitation was not recorded in any case. Review of the endoscopic findings revealed that rectal ulceration was the most common finding seen, being present in 9 (61%) of the cases; in 3 (37%) of these cases there were multiple ulcers. Two patients had rectal polyps, with one of them having multiple polyps [Figure 1a–c]. None of our patients had positive endoscopic findings in areas other than the rectum. Anal fissure was associated with SRUS in 30% of the cases. Two patients had anorectal manometry performed: In one patient the test was reported as normal and the other patient had a nonconclusive test.

**Table 1:** Summary of the clinical finding and the endoscopic features

Age	Sex	Clinical presentation	Endoscopic findings
55	M	Bleeding (r)	Solitary ulcer 12 cm from anal verge
46	F	Constipation, bleeding(r), abdominal pain and mucous discharge	Multiple ulcers 7-8 cm from anal verge
25	F	Bleeding(r), abdominal pain and mucous discharge	Rectal polyp with ulcerated surface
67	F	Constipation, bleeding (r) and abdominal pain	Solitary ulcer 10 cm from anal verge
27	M	Constipation and abdominal pain	Multiple ulcers 20 cm from anal verge
41	M	Constipation, bleeding (r), abdominal pain	Solitary rectal ulcer
27	M	Constipation, and bleeding (r)	Erythematous mucosa 15 cm from anal verge
44	F	Diarrhea	Multiple rectal polyps
33	M	Constipation, and bleeding (r) abdominal pain and mucous discharge	Solitary ulcer 7 cm from anal verge
22	M	Constipation, bleeding (r), and abdominal pain	Thickened mucosa 20 cm from anal verge
85	M	Constipation	Erythema and edema of distal rectum mucosa
27	M	Constipation, bleeding (r) and mucous discharge	Multiple rectal ulcers
15	F	Bleeding (r) and abdominal pain	Solitary ulcer 5 cm from anal verge

(r): Rectal			

### Pathological features

**Figure 2:**
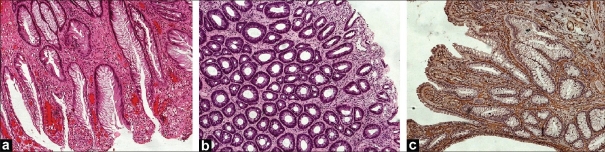
Histological features of SRUS: (a) surface serration with fibromuscular obliteration and crypts' distortion (H and E, ×100); (b) SRUS with diamond crypts (H and E, ×100); (c) immunohistochemical stains for SMA confirming fibromuscular obliteration of the lamina propria

Histological examination of rectal biopsies revealed that the characteristic features were surface serration, crypts' distortion, and fibromuscular obliteration of the lamina propria, and these findings were seen in all cases [Figure 2a]. A specific type of crypt distortion known as diamond crypt was seen in 7 (54.0%) of cases. Diamond crypts are crypts with a triangular (diamond) shape [Figure 2b]. Another common histological feature was ectasia of mucosal vasculature, which was seen in all cases. Five (38%) of cases had congested mucosal vessels. Erosion and ulceration, characterized by discontinuity of the epithelium with acute inflammation and exudates, were seen in 8 (61.5%) of cases. Immunohistochemical stain for SMA highlighted the fibromuscular obliteration of the lamina propria in all the nine cases tested [Figure 2c]. Table 2 summarizes the histological features seen on rectal biopsy in SRUS cases.

**Table 2:** Summary of histological features of the 13 cases of SRUS

Histological features	No. of cases *n*:13	%
Crypts' distortion	13	100
Surface serration	13	100
Diamond crypts	7	54.0
Crypts' branching	3	23.0
Fibromuscular obliteration	13	100
Vascular changes		
Ectasis	13	100
Congestion	5	38.4
Thrombosis	0	0
Hyaline changes	0	0
Inflammation		
Acute	7	54.0
Chronic	10	73.0
Erosion	8	61.5
Immunohistochemistry		
SMA in 9 cases	9	100

## DISCUSSION

SRUS is a rare, benign disorder. The exact etiology is not well understood. One theory is that abnormal rectal evacuation due to paradoxical contraction of the puborectalis muscle may play a role in the etiology of this disease. However, some studies have shown that this paradoxical contraction can be seen in normal individuals also.[7,8] Another etiological hypothesis proposed that there was abnormal defecation due to a reversed pressure gradient produced by the external anal sphincter; however, there is evidence that a normal pressure gradient is present in some SRUS patients.[7,8] A third theory was that trauma and ischemic damage to prolapsed mucosa due to excessive straining may play a role in the pathogenesis.[9,10] The clinical presentation of SRUS is diverse. In addition, endoscopic findings are also varied and can include ulcer, polyp, or only mild erythema. Therefore, SRUS can be a great mimicker of other serious disorders such as carcinoma and inflammatory bowel diseases. In order to study the prevalence of SRUS in our hospital population and to further characterize the clinical and pathological features, we conducted this hospital-based, retrospective study of SRUS in Farwaniya Hospital. Also, we felt that this study will make clinicians and surgical pathologists more aware of this syndrome so that it is less likely to be confused with other conditions.

In the filing system of the Department of Pathology, Farwaniya Hospital, we were able to identify only 13 cases (over a 6-year period) with enough pathological features of SRUS. This indicates that SRUS is a rare disorder. Farwaniya Hospital serves a large (an estimated 800,000) population in Kuwait. In our series, there was a slightly higher proportion of male patients. Chiang *et al*.,[2] in their series of 10 patients, also reported a similar, slight, male preponderance. On the other hand, two other studies have demonstrated a slight female preponderance.[1,11] In our series there was a wide age range (15-85 years). The series from Cleveland Clinic[1] demonstrated a similar wide age range of 14-76 years as also did another study by Marchal *et al*.,[11] which reported an age range of 25-86 years. One of our patients (case 13) was a 15-year-old and can be considered as being in the pediatric age-group. There are only a few reports of SRUS in this age-group in the literature.[12] All patients in our series reported to the hospital because of their clinical symptoms. The triad of rectal bleeding, constipation, and abdominal pain was the most common finding. Rectal bleeding and constipation were the most common presentation in other series also.[1–3,11] The bleeding is likely due to ulceration of the mucosa. Another possibility is that the bleeding was due to associated conditions, such as anal fissure (which was seen in four of our patients) or diverticular disease.[3] Surprisingly, some of the other studies have reported a higher proportion of asymptomatic patients, where SRUS was diagnosed incidentally during colonoscopy done for cancer screening or polyp surveillance. Tjandra reported that 26% of his series were asymptomatic.[1] It is reasonable to expect that patients with SRUS will have constipation, but some patients, unusually, may present with diarrhea. Whereas one of the patients in our series presented with diarrhea, up to 22% of the patients presented with diarrhea in another series.[13] There is no obvious explanation for the diarrhea, but clinicians should be aware of this and consider SRUS in the differential diagnosis in such cases. Rectal digitations and self-inflicted injury have been claimed to contribute to rectal injury,[14] and this has been reported in up to 28% of the patients in some other series.[2] However, none of our patient gave such a history; this could be because ours was a retrospective study and patients usually do not volunteer such information.

Colonoscopic findings are important for the diagnosis of SRUS. Nine (61%) of the patients in our series had rectal ulceration. Some of them had multiple ulcers and some presented with polyps. The lesions were 7-20 cm from the anal verge. Thus, it is obvious that the designation ‘solitary ulcer’ is a misleading. Other series, such as the one published by Torres *et al.*,[13] had similar findings, with 65.3% of the patients reported to have ulceration. In contrast, Tjandra *et al*.[1] reported that 29% of their series had ulcers and 44% presented with polyps. Tendler *et al.*[3] reported that all of his 15 patients had polypoid lesions. Based on these findings, it is obvious that all kinds of rectal lesions can be expected in patients with SRUS, from mild erythema of the mucosa to a solitary ulcer, multiple ulcers, and polyps. Clinicians should be aware of this fact and, in the right clinical setting, should consider SRUS in the differential diagnoses of all kinds of rectal lesions.

Two of our patients underwent an additional test, i.e., anorectal manometry. This test is performed by introducing a manometric catheter to measure the resting and squeeze pressure and the recto-anal reflex in order to discriminate defecatory disorders from other causes of chronic constipation. However, due to the unavailability of this test in our hospital and lack of expertise in interpreting this test, it was performed in only two patients in a different hospital. There are several other investigations that can be carried out in the clinical context of SRUS. Defecography is radiological test used to record anorectal anatomy and pelvic floor motion. In one study,[13] defecography was performed in SRUS patients and several radiological abnormalities were found, including intussusception (70%), rectocele (40%), and internal prolapse and descending prenium (20%). Endoanal ultrasound (EAUS) is also used in SRUS patients who present with chronic constipation to exclude other causes such as sphincter thinning and defects.[15]

Histopathological examination is the key to the diagnosis of SRUS. A combination of fibromuscular obliteration of the lamina propria, crypts' distortion, and surface serration can establish the diagnosis in most cases. Some combination of these features was seen in all the patients in this study. Other authors have also reported that these features are the most common. In the series reported by Tendler *et* al.,[3] crypts' distortion and surface serration was seen in 100% of cases, and fibromuscular obliteration of the propria was seen in 93% of the cases. These changes are seen due to ongoing degenerative–regenerative process occurring in the mucosa. It should be mentioned that these changes can also be seen in inflammatory bowel diseases. However, the absence of other features such as cryptitis, crypt abscess, and granuloma, as well as the clinical setting, can help to differentiate between the two conditions. Diamond-shaped crypts are seen in 54% of cases. Warren *et al*.[4] noticed this feature in all cases of SRUS but not in the ‘control’ cases, such as normal biopsies, irritable bowel syndrome, or adenoma. In other word, the presence of diamond-shaped crypts supports the diagnosis of SRUS but the absence does not totally exclude the diagnosis. Different vascular changes have been noted in biopsies of SRUS. Lonsdale in his series,[5] reported that ectasia with congestion was seen in 95% of cases. Another common feature he noted was muscularized capillaries, which were seen in 50% of his cases. Less common features were thrombosis, fibrin deposition, and atherosis. Tendler and his colleagues,[3] also identified similar mucosal capillary abnormalities, including dilatation, congestion, and thrombosis, in 87% of their patients. Our study revealed similar findings, with ectasia and congestion being seen in 100% and 38.4% of cases, respectively.

In conclusion, this study, to the best of our knowledge, is the first study of this rare syndrome in Kuwait and shows that SRUS in our area has similar clinical and pathological characteristics to SRUS from other areas. This study reaffirms that the SRUS is a misleading term for this condition. The clinical presentation is variable, but the combination of constipation, rectal bleeding, and abdominal pain should alert the clinician to this diagnosis. The endoscopic finding commonly is a solitary ulcer, but other findings can also be seen, such as polyp or erythema. Histological examination is the gold standard for establishing the diagnosis of SRUS. The presence of crypts' distortion, surface serration, and fibromuscular obliteration of the lamina propria are diagnostic features. Clinicians and surgical pathologists should be aware of the features of SRUS so that it is not confused with other conditions.
